# A Multi-Input Neural Network Model for Accurate MicroRNA Target Site Detection

**DOI:** 10.3390/ncrna11020023

**Published:** 2025-03-07

**Authors:** Mohammad Mohebbi, Amirhossein Manzourolajdad, Ethan Bennett, Phillip Williams

**Affiliations:** 1 Department of Computer Science and Information Science, University of North Georgia, Dahlonega, GA 30597, USA; elbenn4041@ung.edu (E.B.); pewilliams@ung.edu (P.W.); 2 Computer Science Department, SUNY Polytechnic Institute, Utica, NY 13502, USA; manzoua@sunypoly.edu

**Keywords:** microRNA target-site detection, deep learning, neural networks, computational biology, bioinformatics

## Abstract

(1) Background: MicroRNAs are non-coding RNA sequences that regulate cellular functions by targeting messenger RNAs and inhibiting protein synthesis. Identifying their target sites is vital to understanding their roles. However, it is challenging due to the high cost and time demands of experimental methods and the high false-positive rates of computational approaches. (2) Methods: We introduce a Multi-Input Neural Network (MINN) algorithm that integrates diverse biologically relevant features, including the microRNA duplex structure, substructures, minimum free energy, and base-pairing probabilities. For each feature derived from a microRNA target-site duplex, we create a corresponding image. These images are processed in parallel by the MINN algorithm, allowing it to learn a comprehensive and precise representation of the underlying biological mechanisms. (3) Results: Our method, on an experimentally validated test set, detects target sites with an AUPRC of 0.9373, Precision of 0.8725, and Recall of 0.8703 and outperforms several commonly used computational methods of microRNA target-site predictions. (4) Conclusions: Incorporating diverse biologically explainable features, such as duplex structure, substructures, their MFEs, and binding probabilities, enables our model to perform well on experimentally validated test data. These features, rather than nucleotide sequences, enhance our model to generalize beyond specific sequence contexts and perform well on sequentially distant samples.

## 1. Introduction

MicroRNAs are small, non-coding RNA molecules of about 23 nucleotides that play a critical role in the post-transcriptional regulation of gene expression [1]. They bind to complementary sequences in the 3’ untranslated regions (UTRs) of messenger RNAs (mRNAs); such binding can lead to translational repression or mRNA degradation [1,2].

The mechanism of microRNA function involves forming the microRNA-induced silencing complex (miRISC) with Argonaut (AGO) proteins. This complex uses the microRNA sequence as a guide to locate complementary target sites on mRNAs and facilitates binding, which can regulate gene expression [2].

Detecting microRNA targets is critical for understanding the regulatory networks that control gene expression and for revealing the functional roles of microRNAs in cellular activities [3,4]. As various diseases such as cancer often arise from irregular gene regulation, microRNAs offer significant therapeutic potential. They can be employed to modulate gene activity and restore normal cellular functions. Furthermore, microRNAs themselves serve as valuable biomarkers for disease diagnosis and prognosis, opening new avenues for personalized medicine [3,4].

MicroRNA targets are identified using various experimental and computational methods. Experimental methods can be broadly categorized into indirect and direct approaches. Indirect methods, such as pSILAC and microarrays, provide a broad overview of potential targets by measuring changes in mRNA expression levels upon microRNA modulation [5,6]. However, these methods lack specificity and cannot pinpoint exact target sites. More focused methods like Western blotting and qPCR, while providing precise gene expression measurements, still cannot identify target sites [7,8]. Luciferase reporter assays offer a stronger experimental approach by directly measuring gene repression upon microRNA binding to a predicted target site [9].

Direct methods, on the other hand, provide more definitive evidence for microRNA-target interactions by either directly detecting the interaction or capturing its products. CLIP-sequencing is a gold standard method that crosslinks microRNA–mRNA complexes, isolates them with AGO antibodies, and sequences the bound mRNAs [10]. AgoTRIBE extends this approach to single-cell resolution by combining AGO proteins with ADAR2 [11]. Degradome sequencing identifies targets by detecting cleaved mRNA fragments [12]. Finally, CLASH crosslinks and ligates interacting RNA fragments, enabling the direct sequencing of microRNA–mRNA interactions [13].

While experimental methods provide solid evidence of microRNA and target interactions, they are costly and time-consuming [14]. Computational methods, by leveraging machine learning algorithms and utilizing experimental data, could provide tremendous help in detecting novel microRNA targets by instantly checking a large set of possible target sites and providing the most likely cases. Computational methods are characterized into two classes: rule-based and data-driven.

Rule-based methods evaluate some predefined features, such as minimum free energy (MFE), base-pairing patterns between microRNA and target site, and site accessibility to detect target sequences. These features are derived based on the current knowledge of microRNA targeting mechanisms [15]. Some of the widely used rule-based tools are miRanda [16], TargetScan [17,18], RNA22 [19], PITA [20], and RNAhybrid [21]. Since these methods perform based on manually selected and limited sets of features, they cannot account for a wide range of microRNA-target types, especially non-canonical target sites. As a result, they suffer from low recall (high false negatives) rates and/or high false positive rates [22,23].

Data-driven methods typically bypass manual feature selection, directly utilizing raw nucleotide sequences as input. Machine learning algorithms then extract and learn nucleotide correlations from extensive experimental datasets [24]. These methods can derive complex correlations between microRNA and target sequences that may not be incorporated in rule-based algorithms. In addition, recent data-driven methods mostly utilize deep learning algorithms [25], which enable these methods to outperform rule-based algorithms. These methods, despite their impressive performance, have two key issues: first, not all nucleotide correlations have a clear biological interpretation, and second, these methods may struggle to be both comprehensive and precise at the same time, often facing trade-offs in sensitivity and specificity. Data-driven methods include DeepMirTar [26], which uses a stacked denoising autoencoder (SdAE) [27]; miRAW [28], which employs an eight-layer Deep Neural Network (DNN) [25]; TargetNet [29], which leverages a ResNet-based deep learning approach [30]; TEC-miTarget [31], which integrates Transformer [32] and Convolutional Neural Network (CNN) architectures [33]; and Mimosa [34], a Transformer-based model.

A major gap in current computational methods for microRNA target site prediction is that, on one side, rule-based approaches suffer from high false positive rates and low predictive power while using biologically interpretable features of microRNA targeting. On the other side, data-driven methods perform better but primarily rely on sequence patterns to achieve their performance. Rule-based methods mainly use features derived from the secondary structure of microRNA target-site duplexes, such as canonical base pairs, and do not incorporate non-canonical base pairs and tertiary structure data, as access to these data is limited. Data-driven methods achieve better performance by learning complex sequence patterns, but they typically treat microRNA and target sequences as natural language strings rather than structured biological entities.

In this paper, we propose a novel framework for microRNA target-site detection that addresses the limitations of computational methods by integrating multiple interpretable data types as input into a unified Multi-Input Neural Network (MINN). Our algorithm processes these independent inputs via parallel CNNs and incorporates the features extracted by the CNNs into a DNN classifier. In addition, we developed a Dynamic Programming (DP) algorithm to predict the binding patterns of the duplex structure between a microRNA and its target site, specific to the domain of these sequences. Our DP algorithm exploits statistical correlations between the two sequences to enhance the contextual relevance of the prediction. The predicted duplex structure is used as one of the inputs for our model. During the structure prediction process, the DP algorithm generates substructures within a duplex, and we provide the scores of these substructures as an additional input. We also compute the minimum free energy (MFE) of these substructures and include them as another input to the model. Furthermore, we generate a probabilistic image, containing the probability of every possible canonical (Watson–Crick) and non-canonical (non-Watson–Crick) base pairs in the duplex structure and use the image as the last input. We train and evaluate our method on an experimentally validated dataset collected from the most reliable resources, representing microRNA target-site interactions.

## 2. Data Collection Procedure

For this study, we utilize two major types of data: (1) probabilities of all possible base pairing between two nucleotides, including canonical and non-canonical pairings, and (2) pair sequences of microRNAs and their binding sites on targeted mRNA genes.

### 2.1. Computing Probabilities of All Possible Base Pairs Between Two Bases

Bases A, C, G, and U pair with each other at different rates due to their structure and the possibility of hydrogen bonds between them. To extract these rates specific to microRNA target-site duplex, ideally, we would need the crystal structure of duplexes, but because of technical challenges, such as the small size of microRNA and target-site duplex, conformational variability of the duplex, and flexibility of its interactions, the 3D structures of these duplexes have not been captured. We found ribosomal RNAs (rRNA) as well-studied biomolecules that are relevant to microRNAs in gene regulation [35], and their crystal structures are available in web databases such as RCSB [36] and RiboXYZ [37]. From RCSB Protein Data Bank (PDB), we extracted 1634 human rRNA structures as PDB/CIF files. Then, by utilizing x3DNA-DSSR software [35,38], we extracted a total of 57,468 canonical and non-canonical base pairs. We computed the probabilities of all possible base pairs, by counting their frequencies in the collected base pairs. These probabilities are shown in Table 1.

### 2.2. Preparing MicroRNA Target-Site Dataset

To have a robust evaluation of microRNA target-site detection methods and a diverse set of microRNA binding duplexes, we used three resources: mirTarBase [39], Helwak et al.’s experimental dataset [13], and Diana-miRBase database [40].

#### 2.2.1. MirTarBase

mirTarBase is a widely adopted database to retrieve MTI information. mirTarBase is updated regularly, and the latest release version 9.0 reported 37 species and contains 2,200,449 curated MTIs. MTIs contained computationally predicted interactions along with experimentally validated targets. For this research, we focus only on experimentally validated MTIs related to human microRNAs. We downloaded the interaction identification (ID) number of the MTIs and then web-scrapped the pages associated with these IDs. From each page, we extracted microRNA and 3’-UTR sequences and stored them in a database. To further improve the quality of our collected dataset, we filtered the MTI entries with strong experimental evidence. Since not all experimental methods provide conclusive information on the target sites of microRNAs, we extracted MTIs that were either explicitly marked in mirTarBase to have strong evidence or supported by research articles that studied microRNA and target-site duplex individually. The purpose of this careful selection of data is twofold: first, to ensure that our model training is free of any possible biases in computational predictions; second, to enhance the credibility of our findings by basing them on high-quality data supported by experiments.

The dataset we collected from mirTarBase consists of 1793 3’-UTR target sequences with 572 microRNAs and 5638 unique interactions between microRNAs and the target genes. MicroRNA lengths were from 22 to 25 nucleotides, and the size of 3’-UTR sequences of their corresponding mRNAs ranged from 54 to 16,211 nucleotides. The average length of 3’-UTR sequences was 2494 with a standard deviation of 2108. To have a fixed-length input for our model, we created fixed-length samples consisting of a 25-nucleotide-long microRNA paired with a 25-nucleotide candidate target site (CTS). Shorter microRNA sequences were padded with space characters.

We defined a sample as positive if the CTS had been experimentally validated as a true target for the microRNA. For every positive microRNA–target-site pair, we generated up to 10 negative samples by the following procedure: First, we randomly select non-overlapping substrings of 25 nucleotides from different parts of the 3’-UTR sequence targeted by the microRNA in the positive sample. Second, we choose only substrings that DuplexFold, a tool from the RNAstructure suite [41] for predicting RNA–RNA binding, predicts a binding between a substring and a microRNA with MFE<0. These negative samples with MFE<0 are challenging for microRNA target-site detection methods because they could occur due to their thermodynamic feasibility yet do not represent true target sites. We want this to help model learn to discern true positives from false positives.

For the cases where the length of 3’-UTR sequences was less than 250 nucleotides, it was not possible to generate 10 non-overlapping negative samples. We also excluded duplicate samples. Given the constraints we applied on the MFE of sample structures and the removal of duplicate cases, we collected 4946 positive and generated 56,120 negative samples. The total number of samples extracted from mirTarBase was 61,066.

#### 2.2.2. Helwak et al. Dataset

Helwak et al. developed the CLASH method, which used for the identification of the target-sites of microRNAs bound to human mRNAs [13]. Their approach provides an unbiased set of microRNA target-site duplexes. Unlike most other experimental approaches, which, in general, use computational predictions or limited data to guide experiments, CLASH directly captures microRNA–mRNA duplexes associated with the AGO protein, one of the major constituents of RISC, thus allowing the detection of both canonical and noncanonical microRNA target sites.

We downloaded the Helwak et al. dataset from the website of the article [13]. It contains records of microRNAs, target sites on 3’UTRs, and their interactions. These interactions were predicted by UNAfold [42]. The interaction data, however, were not used in our training to avoid any bias towards UNAfold’s particular parameters. The target-site lengths vary between 18 and 119 nucleotides, with an average of 52.85 and a standard deviation of 9.37 nucleotides. For each microRNA and target-site pair, we extracted a subsequence with 25 nucleotides starting from the location of microRNA seed binding. With this procedure, we gathered 18,493 pairs of microRNAs and 25-nucleotide target sites. All these pairs are labeled as positive samples.

#### 2.2.3. Diana-TarBase

Diana-TarBase [40] was indirectly used through the miRAW study [28]. We extracted validated negative microRNA and 3’-UTR sequence pairs from the dataset available at this link, which hosts the miRAW dataset and source code. These pairs represent microRNA and 3’-UTR combinations that do not interact. They were originally sourced from Diana-TarBase.

From each of these pairs, we generated up to 10 non-overlapping negative samples by selecting 25-nucleotide subsequences from the 3’-UTR associated with a non-binding microRNA. We filtered out duplications and generated a total of 10,921 negative samples.

#### 2.2.4. Creating Training and Test Sets

We combined the three collected datasets, for which we have a total of 23,439 positive samples, 4946 from MirTarBase and 18,493 from Helwak datasets. The negative samples included 56,120 from MirTarBase and 10,921 from the Diana dataset for a total of 67,041 negative samples. In the final dataset, we have a total of 90,480 samples, including both positives and negatives.

We split the final dataset with the stratified sampling [43] into training, validation, and test sets with ratios of 70%, 10%, and 20%. This method maintains the same ratio of positives to negatives in all the split datasets. The sizes of these sets are as follows: training: 63,334 samples, validation: 9047 samples, and test: 18,096 samples.

## 3. Results

In this section, we outline the training procedure for our model and compare its performance with several widely used microRNA target-site detection methods on our test set. Although numerous microRNA target prediction models have been published, most are either not off-the-shelf tools or are incompatible with our MINN model for several reasons: (1) Limited Scope of Output: Many models, such as miRAW, are designed to estimate the probability of binding between a microRNA and an entire 3’-UTR sequence, rather than identifying precise binding sites. (2) Feature and Length Constraints: Some machine-learning-based methods impose strict requirements on test samples, such as identical feature sets or exact sequence lengths. (3) Obsolete Tools: Several older machine-learning-based methods rely on outdated software libraries or legacy versions that are incompatible with the current development environments, making them inaccessible without significant updates. Given these limitations, and because our MINN model is specifically designed to identify microRNA target sites of a fixed length of 25 nucleotides—equal to the standard size of microRNAs—we selected RNAhybrid, miRanda, RNAduplex, DuplexFold, RNAcofold, TargetScan, RNA22, TEC-miTarget, TargetNet, and Mimosa for performance comparison.

### 3.1. Hyperparameter Optimization and Model Selection

We conducted hyperparameter tuning using a combination of grid search and manual experimentation. For example, for the number of convolutional layers, we experimented with 2, 3, 4, and 5 layers. For filter sizes, we tried multiplications of 2 such as 8, 16, 32, 64, 128, and 256 for each layer. For the dropout rates, we test 0.20 and 0.25 to control overfitting. The optimal number of convolutional layers was three, with filter sizes of 32, 64, and 128. In addition, we experiment with kernel sizes of 2 by 2 and 3 by 3. The optimal was a uniform kernel of 3 by 3. This is consistent with a standard choice for CNNs in image and sequence processing tasks [44].

To observe the training dynamics and behavior of the model across all epochs, we did not use early stopping. Instead, we conducted a grid search over epochs and batch size values and selected the parameter values maximizing the AUPRC of the model on the validation set. We searched over the following value ranges: 5, 20, 30, 40 for the number of epochs and 32, 64, 128, 256, 512 for the batch size. The optimal values found were 20 epochs and a batch size of 256.

**Figure 1:**
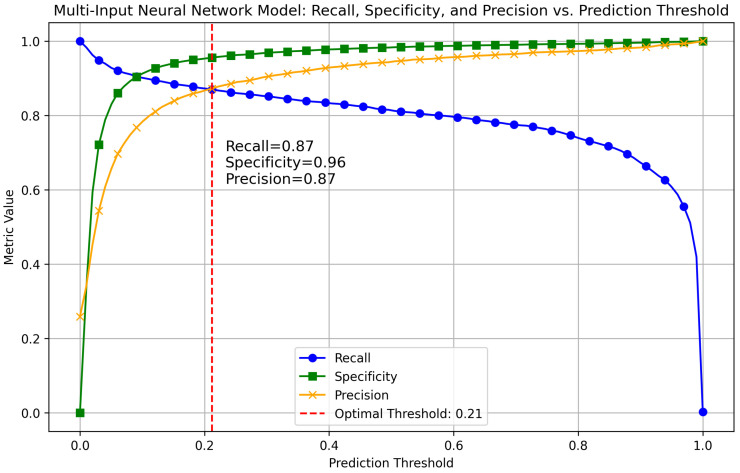
Threshold optimization for our multi-input neural network target-site detection model. Precision, Recall, and Specificity curves are shown for a range of threshold values (0 to 1) to find the optimal threshold to separate target and non-target sites. The optimal threshold was determined by locating the intersection of the Precision and Recall curves, ensuring a balance between these metrics. This figure shows the optimal threshold for our model and the respective Precision, Recall, and Specificity values at the threshold point.

**Figure A1:**
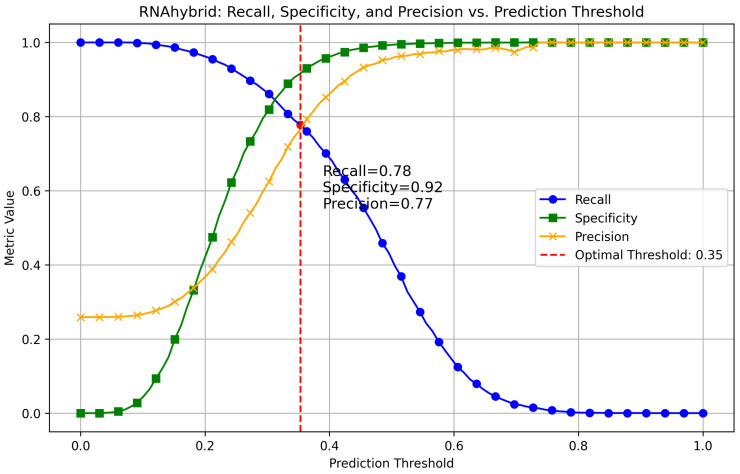
Threshold optimization for RNAhybrid in target-site classification. This figure illustrates the Precision, Recall, and Specificity curves for a range of threshold values (0 to 1) to distinguish target from non-target-sites. Following the same threshold optimization approach as with our model, the optimal threshold for RNAhybrid was identified by locating the intersection of the Precision and Recall curves. Precision, Recall, and Specificity values at this optimal threshold highlight the RNAhybrid’s performance in detecting microRNA target-sites.

To find the optimal threshold for separating target-sites from non-target sites, we looked for a threshold value that simultaneously maximizes both Recall and Precision. We found the optimal threshold by intersecting the Recall and Precision curves, as shown in Figure 1. The figure presents Precision, Recall, and Specificity curves for a range of threshold values from 0 to 1. It highlights the optimal threshold for our model and the values of Precision, Recall, and Specificity at the optimal threshold. We applied the same procedure to other comparative methods; for instance, Figure A1 in Appendix A illustrates the optimal threshold and metric values for RNAhybrid.

The deep learning-based methods we selected for comparison, including TEC-miTarget, TargetNet, and Mimosa, partially used the miRAW method’s dataset for their training and testing. Since the miRAW method uses a CTS length of 40 nucleotides, we adjusted our test set to match this length for a fair comparison. Specifically, for each sample in our test set, we identified its 25-nucleotide CTS within the corresponding 3’-UTR sequences and extracted a 40-nucleotide window with the 25-nucleotide CTS centered in the middle.

### 3.2. Performance Analysis of Computational Methods

**Figure A2:**
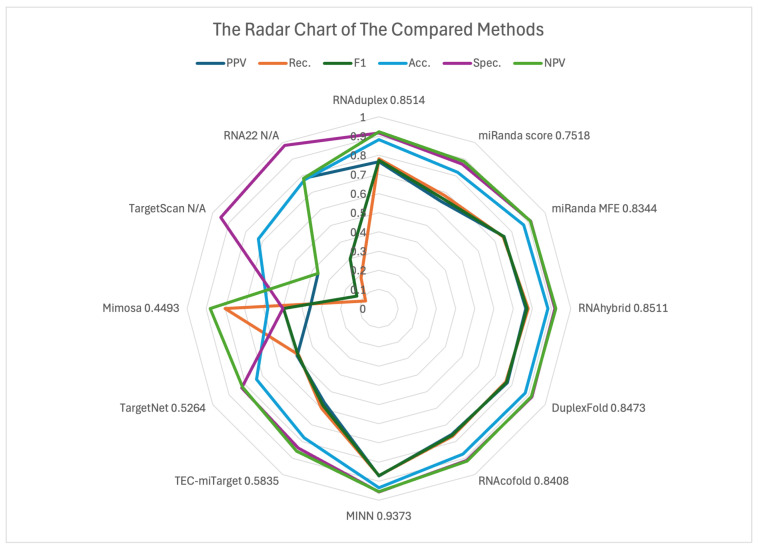
Radar chart illustrating the comparative performance of different methods across key evaluation metrics, including AUPRC, precision (PPV), recall (Rec.), F1 score, accuracy (Acc.), specificity (Spec.), and negative predictive value (NPV). The chart highlights the strengths and weaknesses of each method in predicting microRNA target sites. Note that AUPRC value for each method is placed next to the method name.

In Table 2, we compare the performance of our MINN model versus several computational methods for predicting microRNA target sites, using the aforementioned metrics. Note that the Confusion Matrix results are presented in the Appendix A section, Table A1, and a visual comparison of the methods is provided in Figure A2. The miRanda method provides two ways to distinguish real target-sites: the MFE of microRNA target-site duplex and a score typically ranges from 50 to 200 or higher [16]. We analyze both methods separately for our study, denoted by miRanda MFE and miRanda score. Our proposed model demonstrates the best performance among all methods and surpasses commonly used algorithms. Below is a detailed explanation and comparison of these methods, starting with the highest-performing model and moving down to the least effective one.

Our MINN model provides the best performance on the test set in distinguishing real target sites from non-targets with the highest AUPRC (0.9373), Precision (87.25%), Recall (87.03%), and F1 Score (87.14%). These measures demonstrate a superior performance with a near-optimal balance between Precision and Recall. In addition, the model Specificity (95.55%), NPV (95.47%), and Accuracy (93.35%) indicate that our model is not only precise at detecting true target sites but also is highly capable of correctly identifying true negatives. The robustness of our model minimizes false positives, which is crucial for the experimental validation of microRNA targets and the identification of mRNA targets for a microRNA. In addition, the confusion matrix of our results shows a large number of true positives (4080) and a relatively small set of false negatives (608), highlighting the model’s strength in detecting true microRNA and target-site interactions. The high performance of our model is likely because of training four parallel CNNs, each representing distinctive features and data types, and combining their strength into one classifier. By incorporating structural, base pairing, and free energy information, our model captures the complex dynamics of microRNA–target-site interactions more effectively.

In our performance ranking, RNAduplex ranks second. This method provides a good balance between Precision and Recall, as shown in its F1 Score. It is slightly less specific than our MINN model, but it is still a solid performer in identifying true positive cases. However, compared to our model, it misses more target sites.

RNAhybrid, DuplexFold, miRanda MFE, and RNAcofold performance are very similar to each other with slight variances in the comparing metrics like false positives and false negatives. These methods at their core algorithms use similar thermodynamic principles, such as MFE, to evaluate the binding possibility between RNA sequences. We put them in one category named MFE-based methods. The miRanda score performs weaker than the above methods, achieving a moderate balance between precision and recall.

Deep learning approaches have varying strengths and weaknesses. TEC-miTarget is reasonable in terms of specificity, and very good at detecting true negatives, which makes it effective in transcript-level target predictions, as noted by its authors [31]. TargetNet shows the same trend in the correct classification of true negatives while struggling with Precision and Recall. On the other hand, Mimosa favors Recall over Precision and is, therefore, more suitable for applications which is more costly to miss targets, than identifying false targets. However, its relatively lower Specificity and Accuracy indicate trade-offs that may limit broader applicability.

RNA22 has high Specificity but suffers from low Recall, missing many true interactions. This indicates a conservative strategy that sacrifices sensitivity for higher Specificity. Lastly, TargetScan demonstrates the weakest performance, with minimal Recall and Precision, underscoring its inability to identify a significant portion of canonical microRNA target-site interactions.

### 3.3. Evaluating Generalization Capacity of MINN on an Independent Dataset

To evaluate the MINN model generalization capabilities, we tested its performance on an independent test set, miRAW dataset, a source benchmark extensively used for developing several microRNA target prediction methods such as TargetNet, TEC-miTarget, and Mimosa. This dataset is particularly challenging to evaluate a technique, as it contains negative samples with stable thermodynamic binding (low negative minimum free energy) while they are not actual target site samples. In miRAW work, this dataset was aimed to enhance the miRAW method’s learning capability.

We evaluated MINN on the full miRAW dataset, consisting of 65,000 samples, and compared its performance against several energy-based methods, including RNAduplex, RNAhybrid, miRanda MFE, and miRanda score. Our MINN model achieved an AUPRC of 0.71, significantly outperforming RNAduplex (0.4841), RNAhybrid (0.4829), miRanda MFE (0.4705), and miRanda score (0.5995). This demonstrates MINN’s ability to generalize to an independent dataset with challenging negative samples and highlights its robustness and adaptability. The strong performance of MINN on the miRAW dataset can be attributed to its multi-input model, where it combines information regarding structure, thermodynamics, and base-pairing, and does not rely on one type of information regarding the microRNA binding.

The demonstrated generalization capability of MINN suggests its potential applicability beyond the datasets tested in this study. In future work, MINN can be used to detect microRNA target sites in a range of cell types and disease conditions and enable a deeper understanding of microRNA function in in various biological contexts. In addition, it could be applied in high-throughput microRNA target screening in transcriptomic studies, in which a key challenge is distinguishing between functional targets and non-functional ones. Furthermore, MINN could be integrated with other computational frameworks for microRNA target site annotations in genome-wide analysis or incorporated into hybrid models that combine deep learning with experimental datasets.

### 3.4. Precision–Recall Curves for Method Comparison

**Figure 2:**
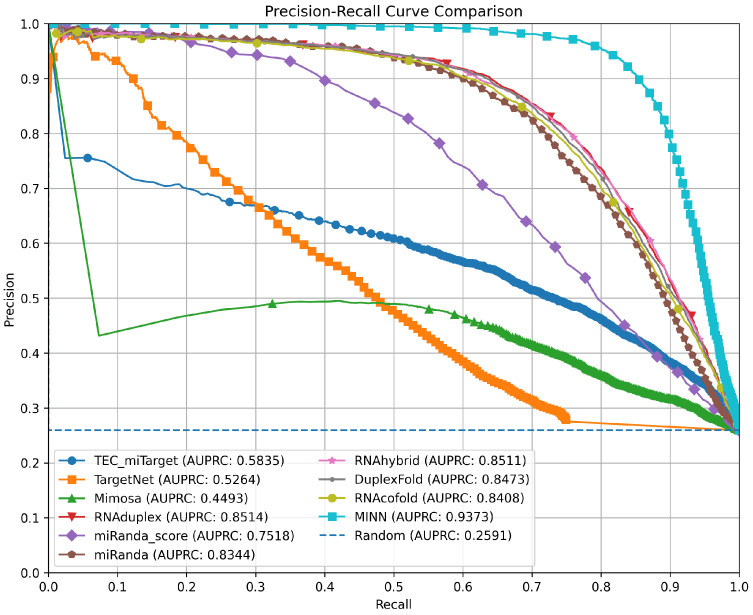
Precision–Recall (PR) curves for our model and the compared microRNA target prediction methods. The curves are generated by sliding a threshold from 1 to 0 in steps of −0.01. The figure illustrates the superior performance of our proposed model compared to others. Notably, energy-based methods exhibit similar performance in the mid-range, while Mimosa, though less effective, still rank significantly above the random classifier line.

For all the compared methods, except RNA22 and TargetScan, we slide a threshold value from 1 down to 0 with the step of 0.01, and compute Precision and Recall metrics at each threshold. The resulting precision and recall values are shown as a PR curve for each method in Figure 2. The figure clearly demonstrates our model’s superior performance. The figure also highlights the similarity in performance among the energy-based methods in the middle range. Mimosa despite exhibiting the lowest PR curve performance still significantly is above the random classifier line. We were not able to compute the PR curve for RNA22 and TargetScan results, since these methods provide binary predictions (0 or 1) without associated probability scores. This limitation prevents us from evaluating the performance of RNA22 and TargetScan across varying thresholds, which would allow for a more detailed PR analysis.

### 3.5. Bootstrap-Based Statistical Comparison of Model Performance

For a true measurement of performance difference between two methods, we need to determine if the difference is statistically significant, or it could have occurred by chance. In this regard, we applied a bootstrap statistical test [45] which, in our case, measures the mean difference in AUPRCs of two compared methods over 1000 resampled test sets. We ran the test using 1000 bootstrap iterations. In each iteration, we resampled from the original test set and computed AUPRC of our model and the compared method. Then, we calculated the mean difference in AUPRC by averaging all the differences in AUPRC that were computed in all iterations. We determined the *p*-value [46] by calculating what proportion of times the AUPRC of our model minus that of the compared model was less than or equal to zero, under the null hypothesis that our model performs no better than the compared model. The associated *p*-value in this case informs us what proportion of the time our model performed no better. For example, a *p*-value of 0.0 provides very strong evidence that MINN consistently yield a higher AUPRC.

To further guarantee that the values did not happen by chance, we used the bootstrapping method to compute 95% Confidence Intervals (CI) [47] of AUPRC. Resampling the test sets 1000 times and recomputing AUPRCs gives us the distribution for AUPRC values, then we selected the middle range enclosing 95% of the distribution. This range forms a robust estimate of the true AUPRC.

The results of our bootstrapping tests are shown in the Table 3. It contains the following columns: Compared method, AUPRC and 95% CI for MINN model, AUPRC and 95% CI for the compared method, mean AUPRC difference, *p*-value, and Percentage AUPRC Difference. The table shows that our model consistently outperforms all the other methods, with higher AUPRC values and statistically significant differences (*p*-value = 0.0). The percentage differences are in range range, from 10.24% versus RNAduplex, to 117.42% versus Mimosa. Mimosa exhibits the largest performance gap compared to our model, while RNAduplex and RNAhybrid demonstrate the closest performance, with smaller differences in AUPRC.

In summary, MINN demonstrates superior performance in predicting microRNA target-site interactions correctly while minimizing false predictions. It outperforms other methods, including MFE-based approaches such as RNAduplex, and deep learning methods like TargetNet, across all the key metrics. While MFE-based methods offer great performance, they are unable balance sensitivity and specificity. Deep learning methods, despite their potential, exhibit average performance in this comparison. We attribute this to the potential sequence variations between these models training data (primarily miRAW) and our diversely collected test set. Methods like RNA22 and TargetScan, which rely on simplified rules, such as seed matching, and site conservation, struggle to predict non-canonical target sites accurately. These findings emphasize the importance of incorporating diverse features, as exemplified by MINN, to understand the complex nature of microRNA target-site interactions.

### 3.6. Logical Basis and Biological Interpretability of Feature Representations in the MINN Model

The MINN model uses four different input matrices, each of which reflects a unique aspect of microRNA–target-site interactions. These features are biologically aligned with the current understanding of microRNA targeting mechanisms. Below, we discuss the biological relevance of each feature and how it contributes to the model’s predictive power.

Since the mechanism of microRNA targeting is not completely known, and the 3D structure of microRNA target site duplex is not available, we tried to capture the reflection of such unknown structure in the four feature matrices that we construct for a duplex. Each feature in the model introduces a specific aspect of microRNA–target interactions. DP-predicted base pairings, including learned biases such as CG bias in the seed and compensatory pairs at 22–24, are stored in a duplex structure matrix. This introduces structural restraints and presents the model with a better reflection of biologically relevant interactions. DP scoring table, in contrast to a duplex structure matrix, stores weights of all possible substructures, compensating for inaccuracies in full duplex prediction by utilizing correct substructures even in the absence of a correct overall structure. Supplementing this, DP MFE table stores information regarding thermodynamic stability, such that DP predictions follow RNA-RNA binding energy laws, critical for detecting functional target site locations.

Additionally, the base pairing probabilities matrix incorporates probability of tertiary interactions extracted from real 3D structures of ribosomal RNAs, and in the process, be able to capture interactions beyond secondary structures, and can therefore detect non-canonical target sites encountered in experimental datasets such as Helwak et al. By combining such rich sets of structural, probabilistic, and energetic information, the model obtains a rich and full picture of microRNA–target-site interactions and can make more accurate and biologically meaningful predictions.

Our DP algorithm plays a critical role in improving the predictive accuracy of the MINN model by predicting microRNA–target-site duplex structures that are specific to the constraints of microRNA targeting mechanisms. Since the 3D structure of microRNA–target-site duplexes is unknown, we rely on a 2D model predicted by our DP algorithm. Unlike generic RNA 2D structure prediction methods, our DP algorithm is tuned to prioritize binding preferences specific to microRNA–target interactions. Importantly, we need to keep in mind that microRNA and target site sequences are guided by the AGO protein structure to bind to each other, and their binding is not like that of two free RNA sequences. These constraints ensure that the predicted structures reflect biologically relevant interactions, which are crucial for accurate target site prediction.

The results of the DP algorithm are utilized in three of MINN’s input channels. First, the Duplex Structure Matrix encodes the complete secondary structure calculated through the DP algorithm. Second, the DP Scoring Table stores all possible substructure weights, which compensate for potential inaccuracies in the prediction of the complete duplex. Third, the DP MFE Table stores minimum free energy values for such substructures, to observe the thermodynamics feasibility of the substructures, ensuring they adhere to the laws of thermodynamics governing RNA–RNA interactions. All three DP-derived inputs together enhance MINN’s ability for biologically relevant microRNA–target-site prediction.

To quantify the contribution of the DP algorithm to the model’s performance, we tested the MINN model with only the DP scoring table (one channel). This configuration achieved an AUPRC of 0.7944 on the validation set, which shows great performance with only one channel. The other channels contribute incrementally to further enhance performance.

#### 3.6.1. Importance of the Duplex Structure Matrix for Capturing Base-Pairing Preferences

The duplex structure matrix contains DP-predicted base-pairing interactions between a microRNA and a CTS. Our DP algorithm predicts the most preferred base-pairing configurations based on the weights learned in the Section 4.1.1. The weights direct the DP algorithm to prefer choosing some base pairs over others in microRNA duplex, such as being biased towards CG pairs in the seed region, as experimentally reported to be essential for target recognition [1,48]. Another example of such preferences is that we observed higher weights for base pairs involving nucleotides 22–24 of the microRNA, which such pairings compensate for mismatches in the seed region in non-canonical target sites [48]. This feature enhances the model performance by providing the model with structural constraints and binding preferences in microRNA–target duplex.

#### 3.6.2. Enhancing Structural Accuracy with the DP Scoring Table

The weights of optimal substructures between subsequences of the microRNA and CTS are stored in the DP scoring table. We use this table as the second input to the MINN. The difference between this table and the duplex structure matrix is that it has the weights of all possible substructures between microRNA and CTS, while the first matrix contains the final DP-predicted base pairs for the entire duplex. This table compensates for the potential errors in the DP algorithm in predicting the entire duplex structure. In such cases, some of these weights correspond to correctly predicted substructures, which could enhance the model to capture correct interactions.

#### 3.6.3. Thermodynamic Insights from the DP MFE Table

The MFE table represents the thermodynamic stability of those substructures with weights that are stored in the scoring table. Thermodynamic stability, as measured by MFE, is one of the key factors in microRNA–target-site binding, as stable interactions are more likely to result in functional repression [49]. By embedding MFE values, the model considers the energy landscape of microRNA–target-site interactions with the guarantee that the predictions will be in line with the thermodynamic principles of RNA–RNA binding.

#### 3.6.4. Base Pairing Probabilities Matrix: Integrating Canonical and Non-Canonical Interactions

This matrix holds probabilities for all possible microRNA and CTS canonical and non-canonical base pairs, derived from structures of ribosomal RNAs (rRNAs). While rule-based methods such as miRanda and TargetScan rely on the secondary structure of microRNA duplex and canonical base pairs for target site prediction, tertiary structure, including non-canonical base pairs, is not yet utilized in such a case. To bridge such a gap, we use non-canonical base pair probabilities from rRNAs to learn non-canonical base pairing patterns in target sites. Target sites, as reported in [13], include non-canonical seed binding, which reflects the diversity of interactions seen in experimental studies of microRNA target sites. This matrix allows every binding possibility to be taken into consideration by the model, enabling it to predict non-canonical target sites that have normally been missed by rule-based methods.

#### 3.6.5. Integration of Features for Enhanced Predictive Power

Our MINN consists of four parallel CNNs. Each of them processes a different kind of input matrix. The CNNs learn unique patterns and interactions specific to their respective channels that capture the structural, thermodynamic, and base pairing probabilistic perspectives of microRNA–target-site interactions. Each CNN learns its hierarchical features independently in a way that the features extracted from each channel are distinct and optimal to their particular input type. The features learned from the parallel channels are then combined, utilizing their different strengths into one powerful model. This combination enables the model to comprehensively learn the binding factors and gives it much stronger discriminative ability between true targets and non-targets with high precision and robustness.

### 3.7. Advantages and Limitations of the MINN Model

The MINN model shows significant improvement over traditional approaches for microRNA target site prediction. The model learns a comprehensive picture of microRNA–target-site interactions through distinct feature matrices capturing structural, thermodynamic, and probabilistic aspects of microRNA targeting mechanisms. MINN can have high performance (AUPRC = 0.9373) with such features. MINN’s generalizability to external datasets, such as miRAW target site dataset (AUPRC = 0.71), shows its strong and flexible performance for a range of challenging microRNA-target site pairs.

Despite its robust performance, MINN has a few limitations. With its use of numerous feature matrices and parallel CNNs, it is computationally intensive, which may limit its use in resource-constrained settings. As with any machine learning model, MINN performance is dependent on training diversity and quality of training data, and it may suffer with datasets that differ significantly from its training distribution. Even though MINN takes structural and thermodynamic features of microRNA targeting, it does not have any explicit consideration for the role played by the AGO protein, a critical participant in microRNA target detection mechanism. The integration of AGO-related features could become a direction for future improvement in model performance and its generalizability.

### 3.8. How the MINN Model Can Be Used and Its Potential Applications in MicroRNA Research

The MINN model can be utilized by sliding a 25-nucleotide window across the 3’UTR of an mRNA sequence to identify microRNA binding sites. The MINN model offers a powerful approach to discovering microRNA functions and regulatory roles. By accurately detecting microRNA target sites on mRNA sequences, the model facilitates the prediction of mRNAs regulated by specific microRNAs. Consequently, this capability enhances exploring microRNA-mediated regulatory networks and their roles in cellular processes. Additionally, since mRNAs are potential therapeutic targets, MINN could be used to research diseases associated with microRNA dysfunction. With its high precision and ease of integration, the MINN model represents a powerful tool for both basic research and translational applications in gene regulation.

## 4. Materials and Methods

In this section, we describe our Multi-Input Neural Network (MINN) model, which learns patterns in distinctive matrices, each representing a different aspect of microRNA duplex structure, and combines the patterns in a deep neural network for classification. These matrices, along with their contents and the computation methods, are explained in the following subsections.

### 4.1. MicroRNA-Specific Secondary Structure Prediction

To our knowledge, there is currently no secondary structure prediction method available, specific to the sequences of microRNA and target-sites. Existing approaches, such as RNAcofold [50], UNAfold, and miRanda, have been used by other researchers to predict these structures. However, these algorithms apply generic RNA-to-RNA binding rules and preferences that may not fully capture microRNA-specific interactions.

Given the influence of AGO protein in the microRNA targeting process [1,51] and insights from experimental studies on microRNA targeting mechanisms [52,53], we hypothesized that these mechanisms may favor certain base pairs in specific regions and exhibit preferences for particular types of base pairs. To capture these preferences, we developed a neural network with a single neuron, where the network inputs (features) represent all possible single, double, and triple canonical base pairs within the duplex. This simplified architecture allows us to extract the weights of input features, which correspond to the expected base-pairing preferences.

#### 4.1.1. Computing Base-Pairing Preferences via a Single-Neuron Neural Network

The six types of canonical base pairs are AU, CG, GC, GU, UA, and UG [54]. We set the maximum length of microRNA and target-site sequences to 25 nucleotides. Each base-pair type is defined as a feature in every possible pairing between a microRNA nucleotide and a target-site nucleotide. The number of features associated with single canonical base pair would be 25×25×6=3750 features.

For double base pairs, we need to consider all possible base pairs in two adjacent pairings. Since there are six types of base pairs, we define 6×6=36 features for each possible case of double base-pairing between nucleotides at positions *i* and i+1 in the microRNA sequence and nucleotides at positions *j* and j+1 in the target site sequence. To optimize the number of features, we only consider the possible double base pairs between index *i* in microRNA and indices [j−2:j+2] in the target site, where j=i. The number of double base-pair features will be 25×5×6×6=4500.

For triple base pairs, we need to have 6×6×6=216 features for each possible case. To reduce the total number of features, we only consider the possible triple base pairs between the nucleotide at position *i* in microRNA and the nucleotides at positions [j−1:j+1] in the target site, where j=i. The total number of triple base-pair features will be 25×3×6×6×6 = 16,200.

**Figure 3:**
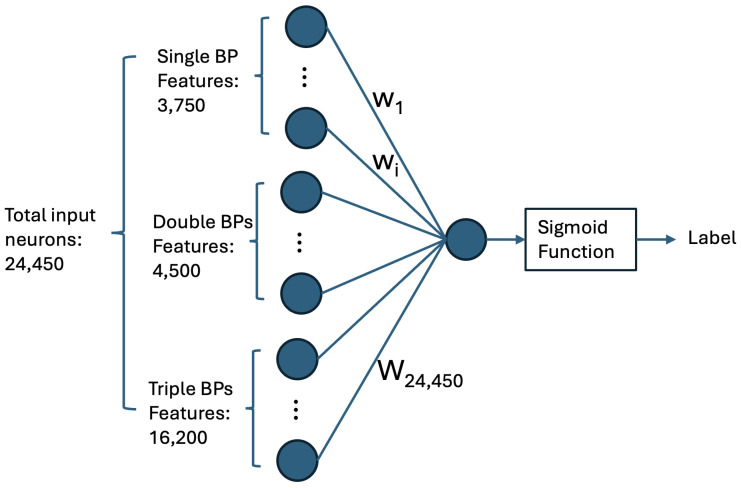
Neural network architecture developed to learn the base pair (BP) preferences of microRNA–target-site duplex structures. This model, with a single output neuron, is constructed using features that represent all possible canonical base pairs (single, double, and triple) between microRNA and target-site nucleotides. The network weights, after training, provide BP preferences in the structure of microRNA–target-site duplex. The weights, resulted from training the model on experimental samples, represent the BP preferences underlying microRNA targeting mechanisms.

The total number of features is 3750 + 4500 + 16,200 = 24,450, and we assign one bit for each feature. For each case of microRNA and target-site sequences, we compute a vector of 24,450 bits, representing all possible single, double, and triple canonical base pairs between the two sequences. Our neural network model has 24,450 input neurons connected to a single output neuron followed by a Sigmoid function. The network is shown in Figure 3.

To understand the significance of each feature, i.e., a single, double, or triple base pair in the duplex of microRNA and its target site, we trained this network for its best possible performance distinguishing target sites from non-target sites and extracted the learned feature weights $w_{1}$ to $w_{24450}$.

We fine-tuned the training of this network for the best AUPRC performance on the validation set and extracted weights of the trained network. We performed a grid search [55] for parameters epochs and batch size and for a range of values of {5,10,20,30,40} and {32,64,128,256} for the parameters, respectively. The optimum parameter values were epochs=5 and batch size=128. The optimum model performance predicting target sites on our test set resulted in an AUPRC of 0.6630, which is decent performance. However, we are not using this model directly for target-site prediction, but rather its feature weights in our DP algorithm for duplex structure prediction.

**Figure 4:**
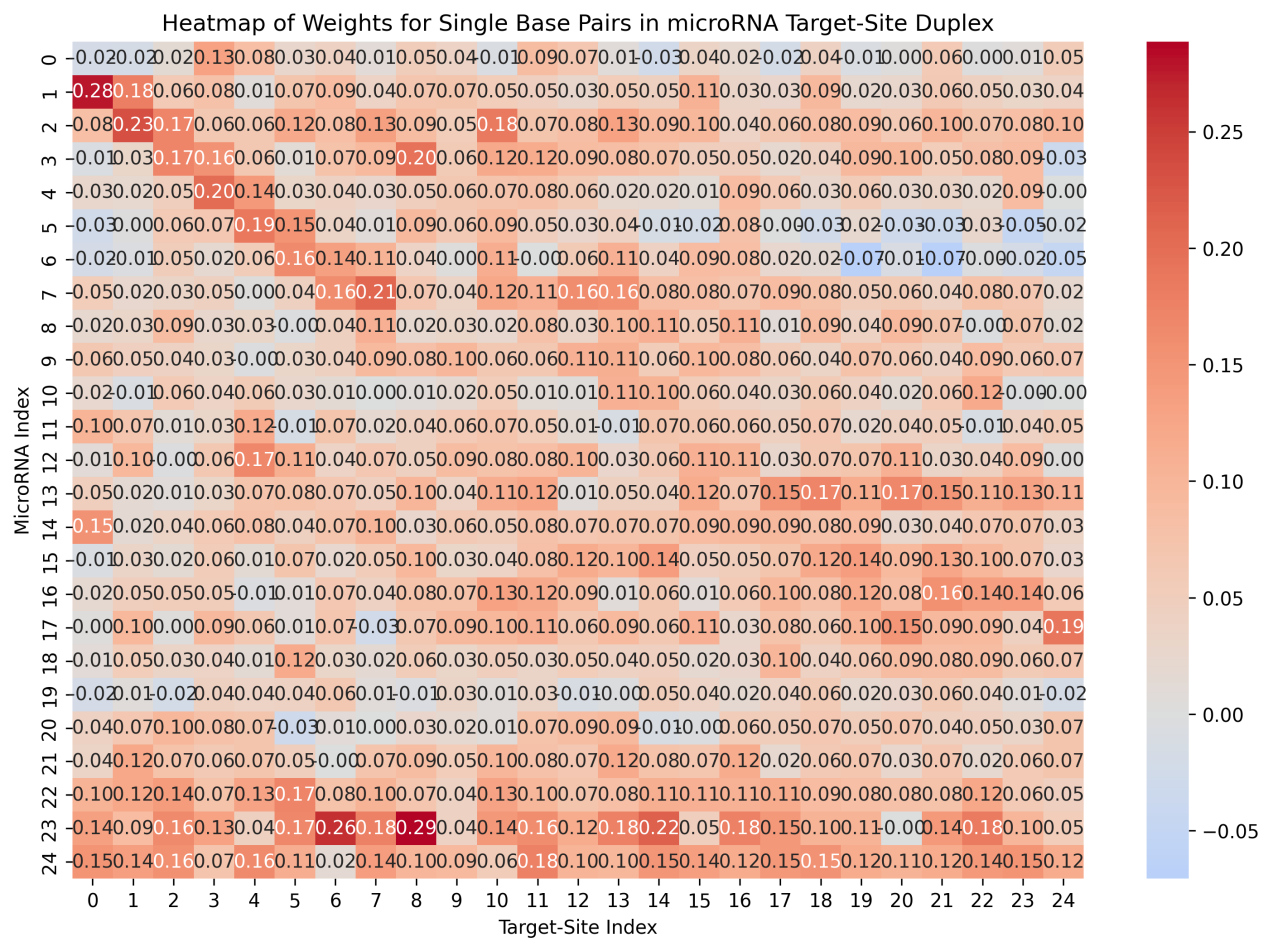
Heatmap of base-pairing weights extracted from the optimal model: This figure shows the model-learned base-pairing weights, indicating a higher preference for pairings in some areas more than others, for example, between microRNA nucleotides 1–7 and target-site positions 0–7. Additionally, nucleotides 22, 23, and 24 of microRNA demonstrate a notable tendency to bind to the target site, which aligns with the experimental findings indicating that base pairing at the microRNA end can compensate for mismatches in the seed region [48].

The weights in the optimal model reveal specific base-pairing preferences that microRNAs exhibit in their targeting mechanisms, as shown in Figure 4. For instance, higher weights in the top-left part of the figure indicate a strong preference for base-pairing between microRNA nucleotides at positions 1 to 7 and target-site indices 0 to 7. Additionally, nucleotides 22, 23, and 24 in the microRNA sequence exhibit a stronger tendency to bind with the target site. These findings are consistent with experimental studies suggesting that base pairing at the microRNA’s 3’ end may compensate for mismatches within the seed region [48].

#### 4.1.2. Distribution of Base-Pair Types in MicroRNA Seed Region

Table 4 presents the percentages of single base pairs within the seed region of microRNAs, derived from the top 100 highest weights in the trained model. The data indicate that GC pairs comprise 44% of the base pairs in this region, while CG pairs account for 16%. Additionally, AU pairs make up 22%, and UA pairs represent 18%, with no GU/UG base pairs initiated by the microRNA seed region. These distributions of base pairs reveal additional underlying patterns that we aim to leverage in predicting the structure of the microRNA target-site duplex, aligning with the patterns observed in experimental samples [56].

#### 4.1.3. Dynamic Programming Algorithm for Duplex Prediction

We develop a dynamic programming (DP) algorithm [57] that exhaustively tries all possible canonical base pairs between the microRNA and the target site to find a structure (set of base pairs) for the duplex, such that the total weight of the structure is maximized. In the following recursion formula of the DP algorithm (1), dp[i][j] represents the maximum total weight possible for a structure between the sub microRNA sequence microRNA[0:i] and the sub-sequence target-site[0:j]. The value of the cell dp[i][j] is the maximum of the following cases:

(a) Ignoring the *i*-th base of microRNA;
(b) Ignoring the *j*-th base of the target-site;
(c) Matching *k* consecutive base pair(s) where k∈{1,2,3}.

The recursion formula is defined as follows: in case (c), the function score_dict() with parameters i−k, j−k, and aligned_bp(s) returns the weights of the aligned base-pairing(s) at indices i−k and j−k, where k∈{1,2,3}. These weights are extracted from the trained model shown in Figure 3.

(1) $$\mathrm{dp}\left[i\right]\left[j\right]=\max\left\{\begin{array}{c} \mathrm{dp}[i-1][j]\;\left(a\right)\\ \mathrm{dp}[i][j-1]\;\left(b\right)\\ \mathrm{dp}[i-k][j-k]+\\ \;\mathrm{score}\text{\_}\mathrm{dict}(i-k,j-k,\mathrm{aligned}\text{\_}\mathrm{bp}(s))\\ \;(\mathrm{for}\;k\in \{1,2,3\})\;\left(c\right) \end{array}\right.$$

Our DP algorithm uses three tables of size 25 by 25: a scoring table S1 for storing the total weights of subproblems, a backtracking table BT1 for traceback and printing the predicted structure of the duplex, and finally, table MFE1 for storing the minimum free energy of the subproblems. These tables are initialized with zero values.

**Figure 5:**
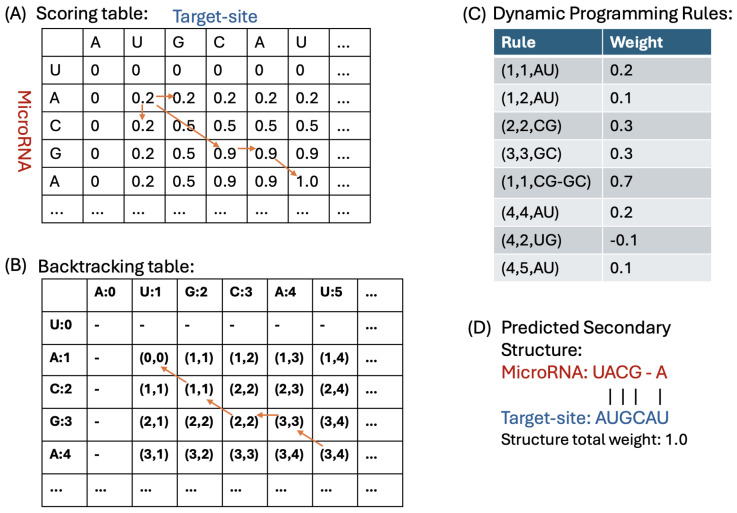
Dynamic programming algorithm for predicting microRNA–target-site duplex structures. (**A**) Scoring table with cumulative weights for microRNA and target-site base pairs, demonstrating how pairing weights accumulate across sequence positions. (**B**) Backtracking table used to trace the optimal base-pairing path, enabling the reconstruction of the predicted duplex structure. (**C**) Dynamic programming rules defining weights for specific base pairs and in particular indices; as an example, rule (1,1,AU) means when microRNA[1] is A, and target site[1] is U, and the weight of such pairing is 0.2. Note that the weights in (**C**) are provided as examples and are not actual values, intended to make the algorithm tables and the figure easier to understand. (**D**) Predicted secondary structure for a microRNA and target-site pair, showing specific nucleotide bindings and the total calculated weight of the structure, reflecting binding preferences based on learned model weights.

**Computing DP table:** We start computing the scoring table from cell [1,1] because, based on experimental studies of microRNA targeting, microRNA[0] and target_site[0] do not pair [52]. To compute cell [1,1], we check for the maximum value among cells [0,1], [1,0], and the value of [0,0] plus the weight of pairing microRNA[1] and target_site[1]. In general, to compute cell [i,j], we check for the maximum value among cells [i−1,j], [i,j−1], and [i−k,j−k] plus the weight of pairing microRNA[i−k:i] and target_site[j−k:j] for *k* values in {1, 2, 3}. The highest value will be stored in score_table[i,j], and the corresponding choice cell, leading to the highest value, will be stored in BT1[i,j]. We compute the MFE of the binding between microRNA[0:i] and target_site[0:j] based on the method presented in the next section and store it in MFE1[i,j]. Figure 5 shows these tables and provides an example of how they are filled and used to predict the secondary structure between microRNA and target-site sequences.

#### 4.1.4. Backtracking and Constructing the Duplex Structure

The backtracking algorithm begins by initializing a pointer at the last cell of the backtracking (BT) table, here at [24, 24]. The next steps depend on the value of this cell, which provides insight into the choices made during the dynamic programming phase. There are two cases:

1. No Pairing Case: If the pointer’s value indicates a transition to either (23, 24) or (24, 23), we move the pointer to one of these cells. In this scenario, it implies that there is no base pairing between the corresponding nucleotides, the microRNA[24] and the target-site[24].
2. Base-Pairing Case: If the pointer’s value is (24−k, 24−k) for *k* values in {1, 2, 3}, it indicates that there are *k* base pairs formed between the nucleotides microRNA[24−k+1:24] and target_site[24−k+1:24]. We record these base pairings and then move the pointer to BT[24−k, 24−k] to continue the backtracking process.

This backtracking process is repeated iteratively from the new pointer location until we reach the starting cell BT1[0, 0]. By following the above procedure and the decisions encoded in the BT1 table, we can reconstruct the optimal duplex structure formed by the microRNA and target-site.

#### 4.1.5. Computing Minimum Free Energy of the Duplex Structure

To compute the minimum free energy (MFE) of microRNA duplex structures, we utilize Turner thermodynamic parameters at a standard physiological temperature of 37 °C [49,58,59]. Since microRNA duplexes have simpler secondary structures compared to large RNA molecules, they only have stacking base pairs, bulges, and internal loops, and the MFE of duplex could be computed by the following Formula (2) [59,60]:

(2) $$\begin{array}{cc} \Delta G_{37}^{{}^{\circ}}= & \Delta G_{37}^{{}^{\circ}}\;\mathrm{intermolecular}\;\mathrm{initiation}\\ \; & +\Delta G_{37}^{{}^{\circ}}(\mathrm{Watson}\text{-}\mathrm{Crick}\;\mathrm{Pairs})\\ \; & +\Delta G_{37}^{{}^{\circ}}(\mathrm{Internal}\;\mathrm{Loop})\\ \; & +\Delta G_{37}^{{}^{\circ}}(\mathrm{Bulge}\;\mathrm{Loop})\\ \; & +\Delta G_{37}^{{}^{\circ}}\;(\mathrm{AU}\;\mathrm{end}\;\mathrm{penalty}) \end{array}$$

Based on Turner thermodynamic parameters, $\Delta G_{37}^{{}^{\circ}}$ intermolecular initiation is +4.09 kcal/mol, and $\Delta G_{37}^{{}^{\circ}}$ AU end penalty is +0.45 kcal/mol if applicable to the structure. The energy change for Watson–Crick pairs, internal loops, and bulges are extracted from Turner parameters [59,60].

In our DP algorithm, we compute the MFE of the optimal substructure between the subsequences microRNA[0:i] and targetsite[0:j], and store it in MFE1[i,j]. The MFE1 represents a matrix of energy changes for all possible sub-duplexes between the microRNA and target site. We use this matrix as an input image in our multi-input model, which is described in the next section.

### 4.2. Multi-Input Neural Network Architecture

To predict the probability of duplex binding between a microRNA (${\mathrm{mic}}_{i}$) and a CTS (${\mathrm{cts}}_{j}$), we develop a multi-input neural network model [44]. For each pair of input sequences—the microRNA ${\mathrm{mic}}_{i}$ and the CTS ${\mathrm{cts}}_{j}$—we calculate four different metrics, as detailed below, and use them as inputs to our multi-input neural network model. In the subsequent steps, we define indices *r* and *c* to represent positions within the microRNA and CTS sequences, respectively. The input matrices for the model are of dimensions 25×25, corresponding to the lengths of the microRNA and CTS sequences.

1. Matrix of Duplex Structure:
  For the input sequences microRNA (${\mathrm{mic}}_{i}$) and CTS (${\mathrm{cts}}_{j}$), we compute a 25×25 matrix. For each index *r* in ${\mathrm{mic}}_{i}$ and each index *c* in ${\mathrm{cts}}_{j}$, we examine whether our DP algorithm’s predicted structure includes a base pair between ${\mathrm{mic}}_{i}\left[r\right]$ and ${\mathrm{cts}}_{j}\left[c\right]$. If a base pair is present, we store the base pair probability in the matrix entry ${\mathrm{MX}}_{1}\left[r\right]\left[c\right]$. This probability is derived from Table 1. The matrix entry ${\mathrm{MX}}_{1}\left[r\right]\left[c\right]$ is filled with zero, if no such base pair is predicted. When ${\mathrm{MX}}_{1}$ is computed, it serves as an image, representing the duplex base pairs and the probability of each pairing, and it is fed to the first channel of our model.
2. DP Scoring Table: For the sequences (${\mathrm{mic}}_{i}$) and (${\mathrm{cts}}_{j}$), our DP algorithm (described in Section 4.1.3) computes a 25×25 scoring table DPs. Each cell DPs[r][c] contains the total weight of the optimal duplex between the subsequences ${\mathrm{mic}}_{i}\left[0:r\right]$ and ${\mathrm{cts}}_{j}\left[0:c\right]$. This table stores the weights of all substructures formed by every possible pair of subsequences.
3. DP MFE Table: Our DP algorithm also computes a 25×25 MFE table, denoted as DPm. For indices *r* in ${\mathrm{mic}}_{i}$ and *c* in ${\mathrm{cts}}_{j}$, cell DPm[r][c] contains the minimum free energy (MFE) of the optimal duplex formed between the subsequences ${\mathrm{mic}}_{i}\left[0:r\right]$ and ${\mathrm{cts}}_{j}\left[0:c\right]$. This table captures the thermodynamic stability of all possible substructures by storing their MFE values, where *r* and *c* represent indices in ${\mathrm{mic}}_{i}$ and ${\mathrm{cts}}_{j}$, respectively.
4. Base-Pair Probabilities Matrix: For the inputs (${\mathrm{mic}}_{i}$) and (${\mathrm{cts}}_{j}$), this matrix captures the likelihood of nucleotide base pairing between the two sequences. Each cell BP[r][c] contains the probability of a base pair forming between the nucleotides ${\mathrm{mic}}_{i}\left[r\right]$ and ${\mathrm{cts}}_{j}\left[c\right]$. These probabilities are derived from Table 1. The BP matrix provides a comprehensive view of the pairing potential across all nucleotide positions in the duplex structure.

We construct a multi-input neural network, that consists of four parallel CNN components, each handling one of the input matrices. These CNNs have identical architecture containing three convolutional layers with filter sizes 32, 64, and 128 and a uniform kernel size of 3 by 3. Convolutional layers use ReLU activation, and each is followed by a max-pooling operation and a dropout regularization to prevent overfitting. The outputs of these CNNs are flattened and merged into a single feature vector.

**Figure 6:**
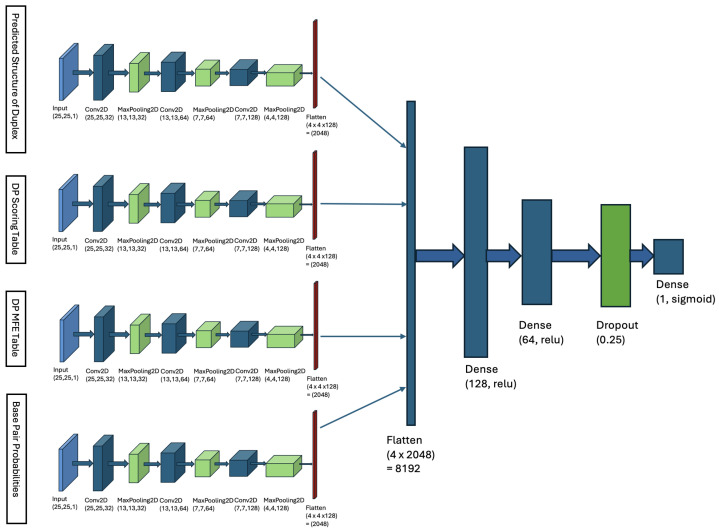
Multi-input neural network (MINN) architecture for detecting microRNA target sites. The model comprises four parallel CNN branches, each processing one of the input matrices. Each CNN has three convolutional layers with filter sizes of 32, 64, and 128, all with a 3 × 3 kernel size. ReLU activation is used in each convolutional layer, and then max-pooling and dropout regularization are applied to avoid overfitting. The outputs from these CNNs are flattened and merged into a single feature vector. This vector is passed through two fully connected layers with 128 and 64 neurons, and one dropout layer with a rate of 0.25. The final layer is a single neuron with sigmoid activation that provides a probability score (between 0 and 1) for the binding chance between microRNA and CTS sequences. This architecture effectively combines multiple inputs to enhance prediction performance.

The feature vector is processed through two dense layers with 128 and 64 neurons, then regulated by a dropout layer with rate 0.25. The final layer is a single neuron with sigmoid activation that provides the probability of binding between microRNA and CTS sequences, as a value between 0 and 1. We refer to this multi-input neural network as the MINN model, and its structure is illustrated in Figure 6.

### 4.3. Evaluation Metrics and Model Comparison

Our metrics for comparison include AUPRC (Area Under the Precision–Recall Curve), Precision, Recall, F1 Score, Accuracy, Specificity, and Negative Predictive Value (NPV). AUPRC measures the model’s ability to balance precision and recall across all thresholds. Its value ranges from 0 to 1, where an AUPRC closer to 1 means a better model performance. AUPRC is a suitable metric for imbalanced datasets where positive samples are the minority class [61]. AUPRC value for a random classifier is the ratio of positives to the total number of samples [62].

Table 5 lists the formulas for these metrics as follows:

- TN (True Negatives): Negative samples predicted correctly.
- TP (True Positives): Positive samples predicted correctly.
- FP (False Positives): Negative samples incorrectly predicted as positive.
- FN (False Negatives): Positive samples incorrectly predicted as negative [63].

We compute AUPRC by approximating the area under the Precision–Recall Curve for a range of n = 100 thresholds, decreasing from 1 to 0 in steps of 0.01. For each threshold $T_{i}$, we calculate the corresponding precision $P_{i}$ and recall $R_{i}$. These values are used in the Formula (3) to compute the AUPRC [61].

(3) $$\mathrm{AUPRC}=\sum_{i=1}^{n-1}\left(R_{i+1}-R_{i}\right)\cdot \frac{P_{i+1}+P_{i}}{2}$$

## 5. Conclusions

In this paper, we proposed a novel algorithm for detecting the target-site of microRNAs. It utilizes various distinctive data points for a microRNA target-site duplex, to build a comprehensive model that detects most of the target-site with high precision. An achievement that most computational methods lack as they choose between having high recall and high precision. Our algorithm includes a Multi-Input Neural Network (MINN) that processes the data points independently via parallel Convolutional Neural Networks (CNNs) and uses the extracted features from CNNs, as input to a Deep Neural Network (DNN) classifier.

Additionally, we designed a microRNA duplex structure prediction algorithm that exploits nucleotide correlations in microRNA and target-site sequences for more domain-specific prediction. The data points we gather for our MINN model are features of this predicted duplex, including the structure, substructures, and the minimum free energy (MFE) values of the substructures. Furthermore, we computed the probability of all possible canonical and non-canonical base pairs in the duplex, based on base-pairing frequencies derived from ribosomal RNA (rRNA) structures. We used the probabilities as pixel values to create a probabilistic image of all possible interactions in a microRNA duplex. This image and the data points previously mentioned were fed to our MINN model.

We constructed a high-quality dataset by collecting microRNA target-site pairs which were experimentally validated, from reliable resources such as mirTarBase, Helwak et al. dataset, and Diana-mirBase. The dataset was split into training, validation, and test sets. We used the training, and validation sets for training and parameter tuning respectively.

Our optimum model yields a superior performance with AUPRC of 0.9373, Precision of 0.8725, and Recall of 0.8703. We compared our results with several commonly used methods including TargetScan, RNAhybrid, miRanda, RNAcofold, RNAduplex, RNA22, and recent deep learning-based models such as TEC-miTarget, TargetNet, and Mimosa. Across all evaluated metrics, our model consistently outperformed these methods.

To ensure our findings are not due to chance, we used the Bootstrap method to compute 95% confidence intervals (CIs) for all compared methods. Our model’s CI did not overlap with the CIs of other methods, indicating the performance difference is statistically significant. Additionally, we used bootstrap hypothesis testing for the null hypothesis that our model performs lower than any other method. The test achieved a *p*-value of zero for all comparisons, this result and the non-overlapping CIs strongly support the superior performance of our model. Our method’s novelty in learning from biologically interpretable features, such as microRNA’s duplex structure, MFE of the substructures, and probability of base pairs, enables it to be more general, and perform well on sequentially different microRNA and target-site pairs. In addition, our method’s superior performance proves it as a valuable tool for biologists to utilize it for microRNA target-site detection. We hope that our method will facilitate research into understanding microRNA’s mechanism in gene regulation, and its application in therapeutic contexts.

## Figures and Tables

**Table 1:** Probabilities of base pair formations in rRNA structures. From 1634 human rRNA structures obtained from the RCSB Protein Data Bank (PDB), a total of 57,468 canonical and non-canonical base pairs were extracted using the x3DNA-DSSR software version 3.0.10. The table lists the computed probabilities of all possible base pair types, based on their frequencies in the dataset.

Base Pair Type	Probability
AA	0.0519
AC/CA	0.0870
AG/GA	0.1566
AU/UA	0.4965
CC	0.0189
CG/GC	0.6979
CU/UC	0.0473
GG	0.0303
GU/UG	0.1210
UU	0.0455

**Table 2:** Performance comparison of computational models for microRNA target-site detection, evaluated at the optimal threshold (Thrs.), using metrics such as AUPRC, Precision (PPV), Recall (Rec.), F1 Score (F1), Accuracy (Acc.), Specificity (Spec.), and Negative Predictive Value (NPV). Note: The threshold for Mimosa method is set to 0.5 by its developers. The results demonstrate superior performance of our proposed MINN model across all metrics.

Method	AUPRC	Thrs.	PPV	Rec.	F1	Acc.	Spec.	NPV
RNAduplex	0.8514	0.3232	0.7659	0.7818	0.7738	0.8816	0.9165	0.9231
miRanda score	0.7518	0.4848	0.6465	0.6815	0.6636	0.821	0.8697	0.8865
miRanda MFE	0.8344	0.2929	0.7538	0.7485	0.7512	0.8715	0.9145	0.9123
RNAhybrid	0.8511	0.3535	0.7656	0.7782	0.7718	0.8808	0.9167	0.9220
DuplexFold	0.8473	0.303	0.7734	0.7643	0.7688	0.8809	0.9217	0.9179
RNAcofold	0.8408	0.3131	0.7577	0.7683	0.763	0.8763	0.9141	0.9186
MINN	0.9373	0.2121	0.8725	0.8703	0.8714	0.9335	0.9555	0.9547
TEC-miTarget	0.5835	0.9899	0.5671	0.5994	0.5828	0.7777	0.8400	0.8571
TargetNet	0.5264	0.4545	0.4913	0.4819	0.4865	0.7365	0.8256	0.8200
Mimosa	0.4493	0.5000	0.3595	0.8020	0.4965	0.5785	0.5004	0.8785
TargetScan	N/A	N/A	0.3660	0.0804	0.1319	0.7257	0.9513	0.3660
RNA22	N/A	N/A	0.7839	0.1849	0.2993	0.7756	0.9822	0.7839

**Table 3:** Results of bootstrapping tests comparing the performance of our model with various microRNA target-site prediction methods. The table includes columns for the method, Area Under the Precision–Recall Curve (AUPRC) and 95% Confidence Interval (CI) for the method. The columns, mean AUPRC difference (Mean Diff.), *p*-value, and the percentage AUPRC difference (% Diff. AUPRC), show the performance difference of the compared method versus our MINN method (* indicating our proposed method). Our model consistently outperforms all other methods, demonstrating higher AUPRC values with statistically significant differences (*p*-value = 0.0). The percentage differences range from 10.24% compared to RNAduplex, to 117.42% versus Mimosa, with Mimosa showing the largest performance gap. RNAduplex and RNAhybrid exhibit the closest performance to our model, with smaller AUPRC differences.

Method	AUPRC	95% CI	Mean Diff.	*p*-Value	% Diff. AUPRC
MINN *	0.9373	[0.9323, 0.9422]	0	0	0.00%
RNAduplex	0.8503	[0.8409, 0.8597]	0.0871	0	10.24%
miRanda score	0.7473	[0.7357, 0.7586]	0.19	0	25.43%
miRanda MFE	0.8343	[0.8246, 0.8436]	0.103	0	12.35%
RNAhybrid	0.8499	[0.8408, 0.8591]	0.0875	0	10.29%
DuplexFold	0.8461	[0.8369, 0.8557]	0.0912	0	10.78%
RNAcofold	0.8395	[0.8298, 0.8498]	0.0979	0	11.65%
TEC-miTarget	0.5801	[0.5657, 0.5965]	0.3571	0	61.58%
TargetNet	0.5245	[0.5106, 0.5386]	0.4128	0	78.72%
Mimosa	0.4311	[0.4187, 0.4456]	0.5058	0	117.42%

**Table 4:** Distribution of Canonical Base-Pair Types in the Top 100 Highest Weights of the microRNA Seed Region: This table shows the prevalence of base pair types, where CG/GC pairs make up 60% (CG 16% and GC 44%), and AU/UA pairs account for 40% (AU 22% and UA 18%). Notably, GU/UG base pairs are absent, indicating that this type of base pair does not initiate in the seed region of microRNAs. These findings suggest location-based base pairing biases that may contribute to the formation of distinctive microRNA target-site duplex structures.

Canonical Base-Pair Type	Percentage
AU	22.0%
CG	16.0%
GC	44.0%
UA	18.0%
UG	0.0%
GU	0.0%

**Table 5:** Evaluation Metrics.

$\mathrm{Precision}=\frac{TP}{TP+FP}$	$\mathrm{Recall}=\frac{TP}{TP+FN}$
$F1\;\mathrm{Score}=2\times \frac{\mathrm{Precision}\times \mathrm{Recall}}{\mathrm{Precision}+\mathrm{Recall}}$	$\mathrm{Accuracy}=\frac{TP+TN}{TP+TN+FP+FN}$
$\mathrm{Specificity}=\frac{TN}{TN+FP}$	$\mathrm{NPV}=\frac{TN}{TN+FN}$

## Data Availability

The MINN source code and datasets are available at: https://github.com/mohebbimg/minn.git.

## Abbreviations

The following abbreviations are used in this manuscript:

AgoTRIBE	Argonaut TRIBE
AGO	Argonaut
CLASH	Crosslinking, Ligation, and Sequencing of Hybrids
CLIP	Crosslinking and Immunoprecipitation
CLIP-seq	Crosslinking Immunoprecipitation Sequencing
CTS	Candidate Target-Site
DNN	Deep Neural Network
DP	Dynamic Programming
DSSR	Dihedral Angle Stepwise Sequence Realignment
miRISC	MicroRNA-Induced Silencing Complex
miRNA	MicroRNA
miTarBase	MicroRNA Target DataBase
mRNA	Messenger RNA
MFE	Minimum Free Energy
MTI	MicroRNA-Target Interaction
PDB	Protein Data Bank
qPCR	Quantitative Polymerase Chain Reaction
RISC	RNA-Induced Silencing Complex
RNAhybrid	RNA-RNA Hybridization Tool
rRNA	Ribosomal RNA
SdAE	Stacked Denoising Autoencoder
UNAfold	Unified Nucleic Acid Folding Algorithm
UTR	Untranslated Region

## Appendix A

### Appendix A.1. Finding Optimal Threshold for RNAhybrid

The optimal threshold for a compared method, as an example RNAhybrid, is the threshold maximizing both recall and precision at the same time, i.e., the intersection of the recall and precision curves. The following figure shows plots of recall, precision, and specificity for RNAhybrid, along with the optimal threshold and the metrics values at the threshold.

### Appendix A.2. Method Comparison

The following table relates to Table 2 and contains Confusion Matrix results for each of the compared methods.

**Table A1:** Confusion Matrix comparison of computational models for microRNA target-site detection, evaluated at their respective optimal thresholds. The Confusion Matrix column represents values as (TP, FN, FP, TN). For the Mimosa method, the threshold is fixed at 0.5 as per its developers.

Method	Threshold	Confusion Matrix (TP, FN, FP, TN)
RNAduplex	0.3232	12,288, 1120, 1023, 3665
miRanda score	0.4848	11,661, 1747, 1493, 3195
miRanda MFE	0.2929	12,262, 1146, 1179, 3509
RNAhybrid	0.3535	12,291, 1117, 1040, 3648
DuplexFold	0.3030	12,358, 1050, 1105, 3583
RNAcofold	0.3131	12,256, 1152, 1086, 3602
MINN	0.2121	12,812, 596, 608, 4080
TEC-miTarget	0.9899	11,263, 2145, 1878, 2810
TargetNet	0.4545	11,069, 2339, 2429, 2259
Mimosa	0.5000	6709, 6699, 928, 3760
TargetScan	N/A	12,755, 653, 4311, 377
RNA22	N/A	13,169, 239, 3821, 867
